# A condensate dynamic instability orchestrates actomyosin cortex activation

**DOI:** 10.1038/s41586-022-05084-3

**Published:** 2022-08-17

**Authors:** Victoria Tianjing Yan, Arjun Narayanan, Tina Wiegand, Frank Jülicher, Stephan W. Grill

**Affiliations:** 1 Max Planck Institute of Molecular Cell Biology and Genetics (MPI-CBG), Dresden, Germany; 2 Biotechnology Center, TU Dresden, Dresden, Germany; 3 Max Planck Institute for the Physics of Complex Systems (MPI-PKS), Dresden, Germany; 4 Center for Systems Biology Dresden (CSBD), Dresden, Germany; 5 Cluster of Excellence Physics of Life, TU Dresden, Dresden, Germany

**Keywords:** Intrinsically disordered proteins, Germline development

## Abstract

A key event at the onset of development is the activation of a contractile actomyosin cortex during the oocyte-to-embryo transition^1–3^. Here we report on the discovery that, in *Caenorhabditis elegans* oocytes, actomyosin cortex activation is supported by the emergence of thousands of short-lived protein condensates rich in F-actin, N-WASP and the ARP2/3 complex^4–8^ that form an active micro-emulsion. A phase portrait analysis of the dynamics of individual cortical condensates reveals that condensates initially grow and then transition to disassembly before dissolving completely. We find that, in contrast to condensate growth through diffusion^9^, the growth dynamics of cortical condensates are chemically driven. Notably, the associated chemical reactions obey mass action kinetics that govern both composition and size. We suggest that the resultant condensate dynamic instability^10^ suppresses coarsening of the active micro-emulsion^11^, ensures reaction kinetics that are independent of condensate size and prevents runaway F-actin nucleation during the formation of the first cortical actin meshwork.

## Abstract (web summary)

A phase portrait study is presented that shows the chemically driven dynamics of short-lived F-actin-rich condensates that support actomyosin cortex formation.

## Main

Morphogenesis involves forces that are generated within the actomyosin cortical layer of cells^1^. Improper cortical organization leads to an impairment of key cellular and developmental processes from as early as meiosis in oocytes to every subsequent cell division^12^. During meiotic maturation of oocytes, the actomyosin cortex transitions from inactive and non-contractile, to active and tension generating^2,3^. This transition can generate a spectrum of actomyosin cortical structures and dynamics, including an actin cap in the mouse oocyte^13^, actin spikes in starfish oocytes^14^ and waves of Rho activation and F-actin polymerization in *Xenopus*^15^. Organizing the first active actomyosin cortex requires the recruitment and assembly of various cortical components as well as the polymerization of actin filaments^4^. These processes have to be coordinated across the entire cell surface in order to generate a uniform actomyosin cortical layer. Here we ask how the formation of an active and tension-generating actomyosin cortex during meiotic maturation in oocytes is orchestrated.

## Actomyosin cortex activation

**Fig. 1:**
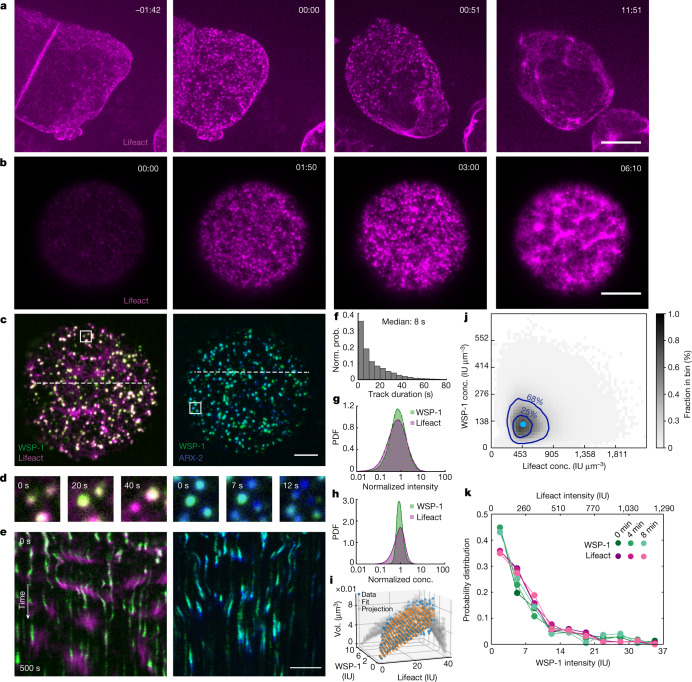
Actomyosin cortex formation at the oocyte-to-embryo transition proceeds through dynamic F-actin/WSP-1 cortical condensates. **a**, In utero microscopy images of the oocyte-to-embryonic transition in *C. elegans* at different times with respect to fertilization (min:s). F-actin (Lifeact::mKate) in magenta; scale bars, 10 μm (**a,b,c,e**). **b**, TIRF images of an isolated oocyte undergoing maturation. In both examples (**a** and **b**), a contractile cortex forms (rightmost image) following a stage characterized by the emergence of short-lived dynamic condensates rich in F-actin (two middle images). **c**, TIRF images of cortical condensates. Endogenous WSP-1::GFP in green (left) and endogenous ARX-2::mCherry in blue (right). **d**, Compositional dynamics of condensates located within the respective white boxes in **c** over time, revealing that adjacent condensates can differ in their instantaneous dynamics. **e**, Condensate dynamics as revealed by kymographs obtained from the white dotted lines in **c**. **f**, Normalized probability (Norm. prob.) of condensate lifetime duration. **g**,**h**, Probability density functions (PDF) of Lifeact::mKate (magenta) and WSP-1::GFP (green) intensities (**g**) and concentrations (conc.) (**h**) within condensates. **i**, Condensate volumes (Vol.) measured (blue) based on the assumption that cortical condensates have a spherical shape and calculated (orange) from the volume dependence on molecular content ${v}_{A}A+{v}_{W}W$. **j**, Instantaneous concentrations of F-actin and WSP-1 within condensates from an ensemble of 36,930 condensates from 9 oocytes. Here 68% and 25% of instantaneous condensate concentrations fall within the outer and inner dark blue contour line, respectively. The light blue dot indicates the peak of preferentially maintained concentration pair of WSP-1 and F-actin in control oocytes. **k**, Normalized probability density functions of WSP-1::GFP (green lines) and Lifeact::mKate (magenta lines); the integrated condensate intensities are similar at 0, 4 and 8 min after the onset of oocyte maturation. Source data

The hermaphrodite nematode *Caenorhabditis elegans* is a prime system for investigating actomyosin cortex formation during oocyte maturation^16–18^. In *C. elegans*, the onset of meiotic divisions and oocyte maturation coincides with ovulation and fertilization^16,18^. Oocytes are fertilized inside the hermaphrodite mother as they pass through the sperm-containing organ—the spermatheca^16^. To understand how the formation of the first actomyosin cortex during oocyte maturation is orchestrated, we visualized F-actin in *C. elegans* oocytes containing Lifeact::mKate2. We observed that, just before fertilization inside the mother, the oocyte cortical layer appears undeveloped with only sparse amounts of filamentous actin present (Fig. 1a, left). By contrast, shortly after fertilization a highly dynamic and dense actomyosin cortical layer is present below the plasma membrane (Fig. 1a, right and Supplementary Video 1). Importantly, we find that actomyosin cortex activation in the oocyte occurs through an intermediate stage that lasts approximately 10 min, results in a dynamic and contractile actomyosin cortical layer, and ends with the extrusion of the first polar body^19^ (Supplementary Video 1). Strikingly, this intermediate stage is characterized by the transient appearance of thousands of F-actin-rich condensates at the cortical layer (Fig. 1a). Here we use the term condensate to refer to a dense assembly of specific molecular components maintained by collective molecular interactions. F-actin and its nucleators have previously been shown to form biomolecular condensates and evidence for liquid-like properties has been provided^5–8^. The F-actin-rich condensates we observe are highly dynamic and inherently unstable. They appear stochastically and each disappear after approximately 10 s.

**Extended Data Fig. 1:**
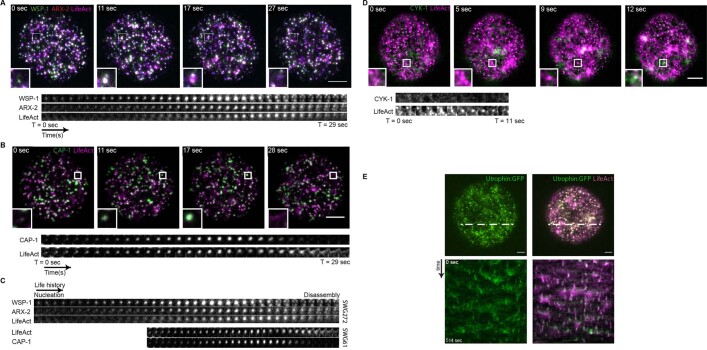
Cortical condensate components. **A**, Top, three-colour imaging of eGFP::WSP1; ARX2::tagRFP and LifeAct::HaloTag; zoomed insets and bottom show a representative cortical condensate example (boxed) together with its time evolution, revealing that the order of arrival of components is WSP-1 followed by ARX-2 and F-actin. **B**, Top, two-color imaging of CAP-1::eGFP; Life-Act::mkate2, zoomed insets and bottom show a representative cortical condensate example (boxed) together with its time evolution, revealing that that CAP-1 comes after F-actin. **C**, Comparing the temporal order of components in **A**, **B** by alignment in time with respect to the maximum LifeAct signal reveals that the order of components recruitment is WSP-1, ARP2/3, F-actin, and CAP-1. **D**, Top, two-color imaging of CAP-1::eGFP; LifeAct::mkate2, zoomed insets and bottom show a representative cortical condensate example (boxed) together with its time evolution. CYK-1/Formin is not present in cortical condensates, but found in contractile actomyosin pulses just prior to large-scale contractions. **E**, Labeling F-actin via Utrophin (left) leads to similar time series as when labeling F-actin via LifeAct (right). Bottom images, respective kymographs determined along dotted white line. Note that in the condition where both LifeAct and Utrophin were imaged (right) colocalization is high between the two channels in the first couple of frames only due to differential bleaching. Scale bars, 10µm.

We next set out to investigate the nature of these transient F-actin-rich condensates. To better observe their dynamics, we took advantage of the fact that oocytes isolated from the mother can mature in the absence of fertilization^20^. This allowed us to develop a total internal reflection fluorescence (TIRF) assay^21^ utilized under highly inclined and laminated optical sheet (HILO) conditions for imaging cellular structures near the cell membrane (Fig. 1b). This enabled a quantitative study of actomyosin cortex formation in isolated oocytes at high spatial and temporal resolution (Supplementary Video 2). F-actin polymerization is organized by nucleation pathway members such as N-WASP and the ARP2/3 complex, as well as the elongator Formin^22^. We first investigated the presence of these components in cortical condensates. We used three strains labelling F-actin (by expressing Lifeact::mKate2) together with either endogenously labelled N-WASP (WSP-1::GFP), capping protein (CAP-1::GFP) or Formin (CYK-1::GFP)^23^. In addition, we used a strain that endogenously labels both the ARP2/3 complex (ARX-2::mCherry) and N-WASP (WSP-1::GFP). As well as F-actin, we identified WSP-1, the ARP2/3 complex and the capping protein CAP-124 (Fig. 1c, Extended Data Fig. 1 and Supplementary Videos 3 and 4) as components of cortical condensates, but the Formin CYK-1 was absent^19,24^ (Extended Data Fig. 1). This demonstrates that cortical condensates contain molecules that mediate branched F-actin nucleation, similar, for example, to CD44 nanoclusters, dendritic synapses and podosomes^25–27^. We also noted that during their approximately 10 s lifetime (Fig. 1f) cortical condensates were enriched first in WSP-1 and ARP2/3, and only then F-actin accumulated before first losing WSP-1 and ARP2/3 and then F-actin (Fig. 1d,e). Given the time at which they appear and the fact that they contain molecules that mediate branched F-actin nucleation, we speculate that dynamic cortical condensates play a role in the formation of the first oocyte cortex.

We next asked if cortical condensates constitute a phase that coexists with its surroundings. Such a phase is characterized by material properties (such as density) that are intensive, that is, independent of volume. We used the strain that simultaneously labels F-actin and WSP-1 to show that, throughout their brief lifetime (Fig. 1e,f), cortical condensates varied over two orders of magnitude in both Lifeact (*A*) and WSP-1 (*W*) integrated fluorescence intensities (Fig. 1g). We estimated the volume of cortical condensates from the cross-sectional area determined by segmentation^28^ (Supplementary Methods), and found that for intensity stoichiometries $A/\left(A+W\right)$ between approximately 0.65 and approximately 0.93, they occupied a volume *V* well described by summing the volume contributions of F-actin ${v}_{A}A$ and WSP-1 ${v}_{W}W$, with volume coefficients ${v}_{A}=1.54\times {10}^{-7}\left(\pm 1\times {10}^{-8}\right)\,{\rm{\mu }}{{\rm{m}}}^{3}\,{{\rm{IU}}}^{-1}$ and ${v}_{W}=2.34\times {10}^{-7}\left(\pm 2\times {10}^{-8}\right)\,{\rm{\mu }}{{\rm{m}}}^{3}\,{{\rm{IU}}}^{-1}$ (where IU denotes total intensity units, see Methods and Fig. 1i). This provides a relation between molecular content and volume, but does not imply that condensates are densely packed structures of only WSP-1 and F-actin. Whereas cortical condensates varied over two orders of magnitude in integrated fluorescence intensities (Fig. 1g), the respective concentrations of WSP-1 and F-actin within the cortical condensates were significantly more restricted in their variation (Fig. 1h). This is also reflected in the emergence of a preferred pair of F-actin and WSP-1 concentrations maintained on average by the ensemble of cortical condensates (Fig. 1j). We conclude that, on the one hand, cortical condensates are maintained far from equilibrium: they are highly dynamic and each disassemble after approximately 10 s. On the other hand, cortical condensates display signatures of a multicomponent condensed phase: they occupy a volume determined by their molecular content and show an enrichment of WSP-1 and F-actin at concentrations distinct from their external environment^29,30^. Hence, the ensemble of stochastically appearing, growing and subsequently dissolving cortical condensates effectively forms a chemically active micro-emulsion, which, despite continuous turnover, maintains a steady size distribution that does not coarsen^11^ (Fig. 1k). Both the properties of a condensed phase and the mechanisms underlying its formation and dissolution can be revealed by a study of growth kinetics.

## Cortical condensate growth laws

**Fig. 2:**
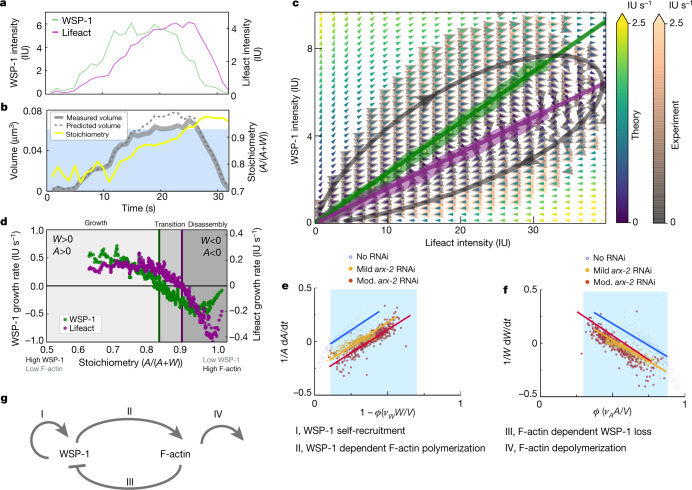
Mass flux phase portrait analysis of cortical condensate growth laws. **a**, Time traces of WSP-1::GFP (green line) and Lifeact::mKate (magenta line) total condensate intensities from a representative cortical condensate. **b**, Time traces of the measured (solid grey line) and determined (dashed grey line) volume using the volume dependence on molecular content ${v}_{A}A+{v}_{W}W$, and stoichiometry $\frac{A}{A+W}$ (yellow line) for the cortical condensate in **a**. The blue shaded region indicates the range of stoichiometry for which the volume dependence accounts for measured volumes (Extended Data Fig. 2). **c**, Mass flux phase portrait measured from 299,165 time points of 36,930 condensates from 9 oocytes (experiment, orange and grey arrows), and calculated from empirically determined growth laws (theory, yellow, green and blue arrows); see Extended Data Fig. 10 for separate representations. The colour scale denotes time rate change vector magnitudes. Thick lines indicate WSP-1 (green) and F-actin (magenta) nullclines from experiment; thin lines indicate theoretical nullclines. Absolute molecular amounts can be estimated for WSP-1 with 8 IU corresponding to approximately 100 WSP-1 molecules. **d**, Measured WSP-1 (green) and F-actin (magenta) growth rates as a function of stoichiometry display three regimes separated by the WSP-1 nullcline at stoichiometry approximately 0.85 and the F-actin nullcline at stoichiometry approximately 0.9. **e**,**f**, Linear dependence of relative WSP-1 (**e**) and F-actin (**f**) growth rates—in the unperturbed control (blue) and mild *arx-2* RNAi (orange) and moderate (mod.) RNAi (red) cases—on effective F-actin volume fraction *ϕ* (Supplementary Information). Linearity holds within the blue shaded region (see **b**, Extended Data Fig. 2) and is indicated with lines, yielding the parameters *k*_r_, *k*_l_ (**e**) and *k*_b_, *k*_d_ (**f**). **g**, Reaction motif underlying the structure of **c**–**f** composed of WSP-1 self-recruitment, WSP-1 dependent F-actin polymerization, F-actin dependent WSP-1 loss and F-actin depolymerization. Source data

To study the growth kinetics of these cortical condensates, we quantified their compositions and volumes over time (Fig. 2 and Supplementary Methods). For a single representative cortical condensate, Fig. 2a,b shows the time evolutions of (1) WSP-1 and F-actin total condensate intensity, (2) stoichiometry and (3) volume (Supplementary Notes 1 and 2). For the example shown, WSP-1 precedes F-actin in both growth and loss, stoichiometry grows monotonically with time, and volume first increases and then decreases, and is well captured by summing volume contributions from F-actin and WSP-1. We noted that neighbouring condensates followed similar trajectories in composition and volume despite forming stochastically and at different times (Fig. 1c–e and Extended Data Fig. 9). Thus, at a given time, neighbouring cortical condensates that share their external environment can be at different stages of their internal life cycle. We conclude that the growth kinetics postnucleation are governed by condensate internal composition.

How does the internal composition of a cortical condensate influence its growth and shrinkage? To answer this question we developed a general method to quantitatively study compositional dynamics in an ensemble of multicomponent condensates based on an analysis of the mass flux into the condensates (mass balance imaging^31^). For this, we quantified the time rate change of protein amounts within cortical condensates as a function of their internal F-actin and WSP-1 amounts. This time rate change of amounts is represented by a vector field, which defines average trajectories in the space of WSP-1 and F-actin amounts (Fig. 2c). Consistent with the representative example (Fig. 2a), average trajectories form loops that pass through three subsequent regimes: an early growth regime in which condensates first grow in WSP-1 and subsequently in F-actin amounts; a transition regime in which WSP-1 is lost while F-actin amounts still increase; and a disassembly regime with loss of both WSP-1 and F-actin. The nullcline of WSP-1 dynamics (the green line in Fig. 2c), that is, the WSP-1 amounts above which condensates grow and below which they shrink in WSP-1 content, reflects an F-actin-dependent critical WSP-1 amount for WSP-1 growth. Stoichiometry is constant on lines that pass through the origin, and hence the WSP-1 nullcline corresponds to a threshold stoichiometry of approximately 0.85. F-actin growth dynamics change from growth to shrinkage at a similar but slightly higher stoichiometry of approximately 0.9 (the magenta line in Fig. 2c shows the F-actin nullcline). We conclude that cortical condensates become unstable and change from growth to disassembly in the transition regime between the two nullclines.

The three regimes (growth, above the WSP-1 nullcline; transition, between the two nullclines; disassembly, below the F-actin nullcline in Fig. 2c) are also visible when plotting WSP-1 and F-actin growth rates as a function of stoichiometry (Fig. 2d). Because the stereotypical compositional trajectories (Fig. 2a,b) involve a monotonic increase in stoichiometry with time, the *x* axis of Fig. 2d also represents a progression through time. The dependence of growth rates on stoichiometry reveals the mutual regulation of WSP-1 and F-actin, and can be depicted by the reaction motif shown in Fig. 2g. Processes I and II are mediated by WSP-1, whereas processes III and IV are mediated by F-actin. Process I corresponds to WSP-1 self-recruitment, evidenced by the fact that at low stoichiometry, corresponding to condensates consisting of mainly WSP-1, the WSP-1 growth rate is largest (Fig. 2d). Process II denotes WSP-1 dependent F-actin growth, reflected by a decrease of the F-actin growth rate as stoichiometry increases. This is most evident in the transition regime of Fig. 2d. Process III denotes F-actin-dependent loss of WSP-1, reflected by the fact that WSP-1 growth rates decrease with increasing stoichiometry. This suggests that F-actin counteracts the ability of WSP-1 to self-recruit, similar to previously reported negative feedback of F-actin on its nucleation via Rho^15,32^. Finally, process IV denotes F-actin depolymerization, reflected by the fact that F-actin is lost fastest at the highest stoichiometry (Fig. 2d). Further support for this reaction motif is provided by an analysis of WSP-1 and F-actin growth rates at constant WSP-1 and F-actin amounts (Extended Data Fig. 9), and an analysis of the impact of RNA interference (RNAi) of proteins involved in regulating F-actin (Extended Data Fig. 4).

**Extended Data Fig. 3:**
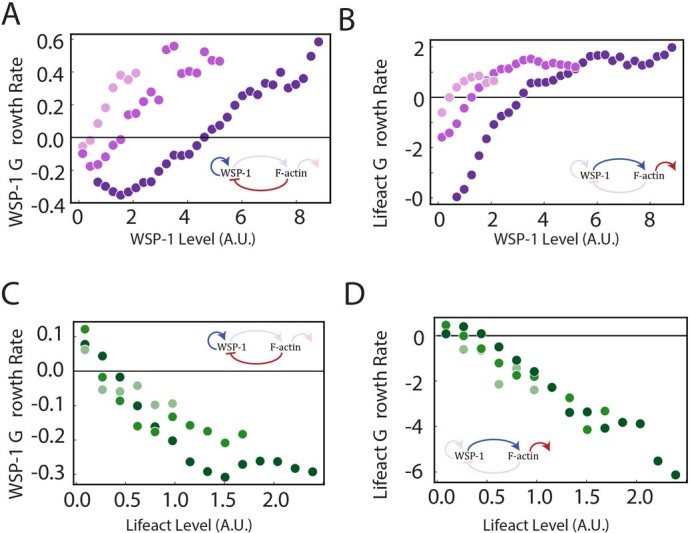
Mass flux phase portrait slices corroborate the empirical growth laws. **A**,**B**, WSP-1 (**A**) and F-actin (**B**) growth rates as a function of WSP-1 at constant levels of LifeAct (increasing darkness of dot color represents the first, second and third lowest lifeAct bins of Fig. 2c, respectively). **C**,**D**, WSP-1 (**C**) and F-actin (**D**) growth rates as a function of F-actin at constant levels of WSP-1 (increasing darkness of dot color represents the first second and third lowest WSP-1 bins of Fig. 2c, respectively). Insets in **A-D** indicate contributing feedback processes (Fig. 2g). Source data

The shape of the measured phase portrait (Fig. 2c) and the shape of the growth rates $\dot{W}$ for WSP-1 and $\dot{A}$ for F-actin as a function of stoichiometry (dots denote time derivatives; Fig. 2d) suggest the following empirical growth laws that define a non-linear dynamical system^33^ (Fig. 2c, Extended Data Fig. 3 and Supplementary Note 3):

$$\begin{array}{l}\dot{W}={k}_{{\rm{r}}}W-{k}_{{\rm{l}}}\frac{AW}{V}\\ \,\dot{A}={k}_{{\rm{b}}}\frac{AW}{V}-{k}_{{\rm{d}}}A.\end{array}$$

Here WSP-1 self-recruitment depends linearly on *W* through the recruitment rate *k*_r_, consistent with the ability of WSP-1 molecules to dimerize^34,35^ (process I). Interactions between F-actin and WSP-1 result in ARP2/3 mediated branched nucleation and a subsequent increase in the amounts of F-actin^36,37^. This behaviour is captured by the term ${k}_{{\rm{b}}}\frac{AW}{V}$, where *k*_b_ is a kinetic coefficient describing branching and condensate volume $V={v}_{A}A+{v}_{W}W$ depends on molecular amounts (see above and also Supplementary Information; process II). Branched nucleation coincides with a loss in WSP-1; this loss is captured by ${k}_{{\rm{l}}}\frac{AW}{V}$ with the kinetic coefficient *k*_l_ describing the branching-dependent loss of WSP-1 (ref. ^38^; process III). Finally, F-actin is lost with rate *k*_d_, consistent with severing and depolymerization^39^ (process IV) (see also the simplified depiction in Extended Data Fig. 6). Note that these four coefficients together capture all the relevant molecular processes inside the condensates. This may include processes not discussed above. The mathematical form of all four terms is determined by the observation that the relative growth rates $\dot{W}/W$ and $\dot{A}/A$ are linear functions of the effective F-actin volume fraction $\varphi =\frac{{v}_{A}A}{V}$ (Fig. 2e,f; see also discussion in Supplementary Note 3). Figure 2e,f also allow us to estimate *k*_r_, *k*_l_, *k*_b_ and *k*_d_. With these estimates, the simple growth laws describe the experimental data well, and capture the entire mass flux phase portrait together with the composition-dependent critical sizes as reflected by nullclines (Fig. 2c).

The WSP-1 nullcline ${W}_{{\rm{c}}}(A)=A({k}_{{\rm{l}}}-{k}_{{\rm{r}}}{v}_{A})/{k}_{{\rm{r}}}{v}_{W}$ specifies a critical amount of WSP-1 above which WSP-1 amounts grow and below which WSP-1 amounts shrink. Notably, this critical amount is similar to a critical droplet size for nucleation and growth, but here it stems from biochemical reactions and not from condensation physics. The resulting growth laws exhibit a fixed point at $\left(A,W\right)=\left(0,0\right)$ with a stable (F-actin) and an unstable (WSP-1) direction. After nucleation, condensate dynamics follow a homoclinic orbit, initially growing rapidly in the unstable direction before turning and eventually undergoing disassembly while moving along the stable direction back towards the fixed point. Together, this represents a dynamic instability of condensates that shares similarities with the dynamic instability of microtubules^10^: cortical condensates transition from unstable growth to shrinkage, which limits their size, and can display stochastic rescue events (Extended Data Fig. 7).

## Transition to unbounded growth

**Fig. 3:**
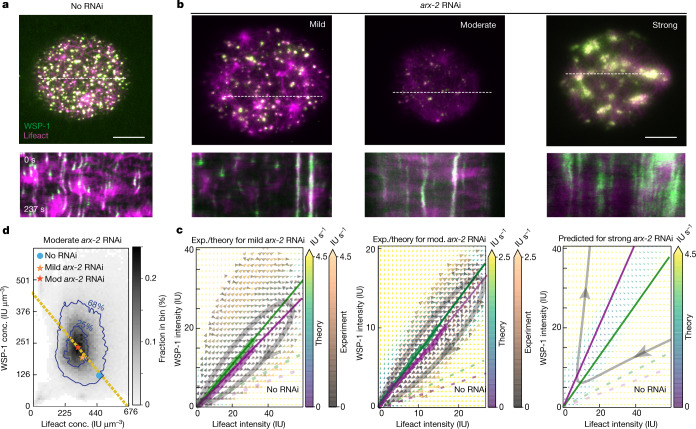
ARX-2 controls ensemble WSP-1/F-actin concentrations inside condensates by tuning condensate dynamics. **a**, Top, TIRF image of cortical condensates in an unperturbed control oocyte. Bottom, kymograph (determined along the dotted white line) revealing temporal dynamics. **b**, Top, TIRF image of cortical condensates under mild, moderate and severe *arx-2* RNAi (left to right). Mild and moderate *arx-2* RNAi datasets corresponded nominally to 18–20 and 19–20 h of *arx-2* RNAi, respectively, and were characterized by different numbers of condensates in the steady state (Supplementary Information). Bottom, respective kymographs (determined along the dotted white lines) revealing temporal dynamics. Scale bars, 10 μm (**a**,**b**). **c**, Experimental (exp., orange and grey arrows) and theoretical (theory, yellow, green and blue arrows) mass flux phase portrait of mild and moderate *arx-2* RNAi oocytes (left and centre) and predicted severe *arx-2* RNAi phase portrait (right) using *k*_d_ estimated from the progressive change in *k*_d_ from control to moderate RNAi datasets (Extended Data Fig. 7 and Supplementary Notes). Colours denote time rate change vector magnitudes. Thick lines indicate the measured WSP-1 (green) and F-actin (magenta) nullclines; thin lines indicate theoretical nullclines. Dashed lines indicate nullclines from unperturbed control oocytes (Fig. 2c; Extended Data Fig. 10b shows nullclines in a single graph). The representative streamline is shown in grey. **d**, A histogram of instantaneous concentrations of F-actin and WSP-1 within condensates for moderate *arx-2* RNAi. Here 68% and 25% of instantaneous condensate concentrations fall within the respective blue contour lines. The blue dot and orange and red stars represent the preferentially maintained concentration pair for the ensemble of control, mild and moderate *arx-2* RNAi oocytes, respectively. Yellow dashed line: line of constant total density given by volume relation. Source data

**Extended Data Fig. 4:**
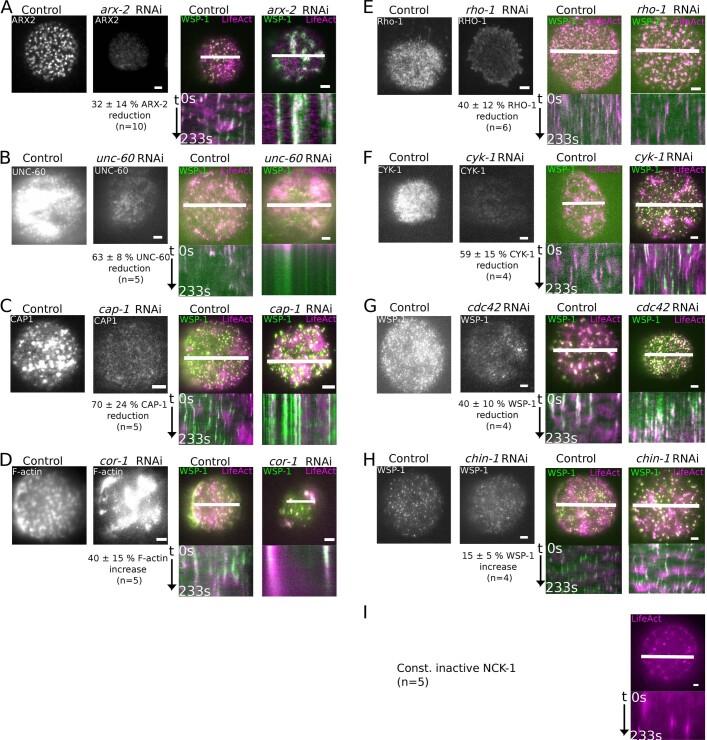
RNAi perturbations of cortical condensates. **A-H**, Left two panels, quantification of the efficacy of 24 h of RNAi via fluorescence imaging (right) and a quantification of the relative change in protein levels with respect to the non-RNAi control (left). Right two panels, WSP-1 and LifeAct fluorescent images of cortical condensates in the non-RNAi control (left) and after 24 h of RNAi (right) together with respective kymographs (bottom) determined along the white lines. **A-D**, 24h of RNAi directed against ARX-2, UNC-60, CAP-1 or COR-1 leads to stable large patches of WSP-1 and F-actin. In the case of COR-1, RNAi efficacy is reflected in a ~40% increase of F-actin at the cortex. **E**-**H**, 24hrs of RNAi directed against RHO-1, CYK-1, CDC42 or CHIN-1 does not appear to affect cortical condensates. In the case of RNAi directed against CDC42 and CHIN-1, RNAi efficacy is reflected in a ~ 40% and a ~ 15% increase in WSP-1. **I**, Dynamic cortical condensates persist for a strain with constitutively inactive NCK-1.

**Extended Data Fig. 6:**
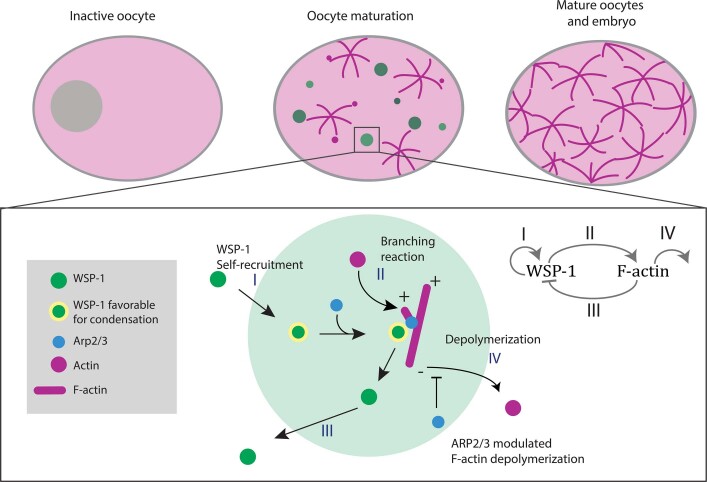
Coarse-grained schematic suggested by growth laws. Schematic of oocyte cortex formation through an intermediate stage with WSP-1, ARP2/3 complex, and F-actin condensates. The four processes that underly the dynamic instability of condensates are WSP-1 self-recruitment, branching-dependent WSP-1 loss, branching-dependent F-actin growth, and F-actin loss (depolymerization or severing modulated by ARP2/3). The effect of *arx-2* RNAi on the F-actin loss coefficient is indicated. As the rate coefficients reflect the summed effect of all processes affecting composition dependent condensate growth molecular mechanisms are likely to be more involved, but maintain the depicted overall structure.

**Extended Data Fig. 7:**
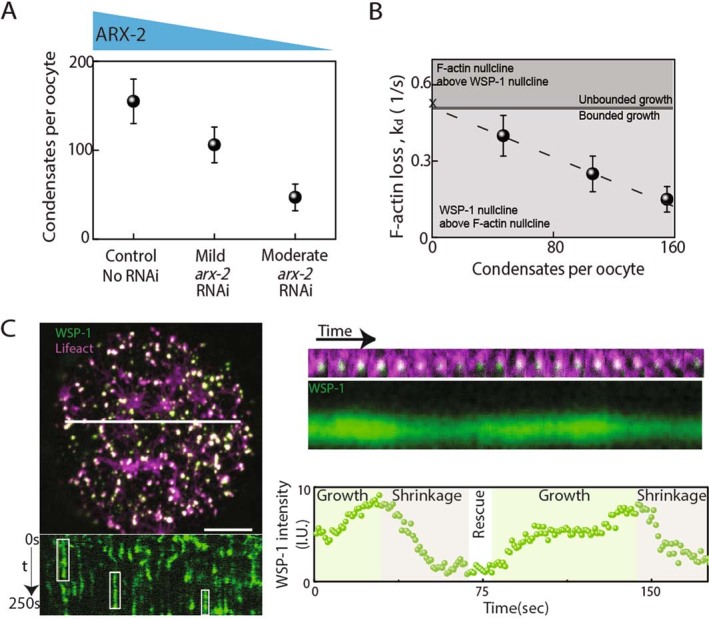
Transition to unbounded growth, and rescue events. **A**, The steady state number of condensates per oocyte in non-RNAi control oocytes and in oocytes subjected to 18-20hrs of *arx-2* RNAi was used to classify RNAi phenotype severity - mild (between 30 and 70 cortical condensates per oocyte) and moderate (between 70 and 120 cortical condensates per oocyte) *arx-2* RNAi. **B**, Non-RNAi control, mild and moderate *arx-2* RNAi conditions as described in **A** showed a systematic increase in *k*_*d*_ with a decrease of the number of cortical condensates per oocyte. In **A**, **B** the control, mild and moderate datasets comprised *n* = 9, *n* = 7 and *n* = 7 oocytes respectively while the error bars correspond to standard errors of mean (**A**) and the margin of error of a 95% confidence interval (**B**) respectively. Severe *arx-2* RNAi leads to a loss of dynamic cortical condensates (with zero condensates per oocytes) and the emergence of large persistent patches of WSP-1 and F-actin, in agreement with unbounded growth (Fig. 3b, right). Hence, we expect the value of *k*_*d*_ at zero cortical condensates per oocyte to coincide with the critical value of *k*_*d*_ at which condensate dynamics become unbounded. A linear fit (dashed line) provides an extrapolated estimate of the y-intercept value of *k*_*d*_ = 0.49 ± 0.04. Note that this value is similar to the critical value of *k*_*d*_ ≈ 0.47 at which the nullclines switch position (with the three other growth coefficients determined from moderate *arx-2* RNAi), with the A-nullcline now above the W-nullcline (see Fig. 3c, right; compare to left, middle). In the graph above, the x on the y-axis marks the value of *k*_*d*_ = 0.51 used for calculating the phase portrait for severe *arx-2* RNAi (Fig. 3c, right), which is in the unbounded growth regime. **C**, Left, rescue events in the representative image shown in Fig. 1c. Top, snapshot; bottom, kymograph along white line showing rescue events (indicated by green boxes). Right, one representative rescue event of a cortical condensate showing two cycles of growth and shrinkage, separated by rescue. Top, time-series; middle, kymograph; bottom, integrated WSP-1 intensity. Scale bar, 10μm. Source data

To understand how the transition from condensate growth to condensate disassembly is orchestrated, we used RNAi to perturb the interplay between WSP-1 and F-actin. RNAi of upstream signalling molecules that regulate F-actin assembly, such as RHO-1 (Rho GTPase), CYK-1, CDC-42 and CHIN-1 (CDC-42 GAP), as well as multivalent adaptors VAB-1 (Ephrin receptor) and NCK-1 (Nck) did not affect condensate dynamics^40–42^ (Extended Data Fig. 4 and Supplementary Notes 4 and 7). This suggests that cortical condensate dynamics are governed by feedback structures independent of the major signalling pathways that regulate the actomyosin cortex^43^. WSP-1 mediates branched F-actin nucleation through the ARP2/3 complex^4,35^, and we thus performed RNAi against ARX-2 (ARP2 in the ARP2/3 complex in *C. elegans*^44^). Oocytes showed reduced numbers of cortical condensates for less than 20 h of *arx-2* RNAi, whereas cortical condensates were absent for more than 20 h of RNAi (Fig. 3a,b and Extended Data Fig. 7A), consistent with a general reduction of F-actin branched nucleation^45^.

We used the reduction in the number of cortical condensates as a measure of the strength of the perturbation, and distinguished between mild (30 to 70 condensates per oocyte), moderate (70 to 120 condensates per oocyte) and strong *arx-2* RNAi (no dynamic cortical condensates). Mass balance imaging of cortical condensates in the mild and moderate conditions revealed that, in comparison with the unperturbed case, compositional trajectories are progressively tilted towards the WSP-1 axis (Fig. 3c). The mathematical form of the growth laws is maintained, but the associated coefficients are changed (Fig. 2e,f). Mild and moderate *arx-2* RNAi reduced the rate of WSP-1 self-recruitment *k*_r_ by 17 ± 4% and 15 ± 4%, respectively, and increased the coefficient *k*_l_ for moderate *arx-2* RNAi by 20 ± 6% (1 ± 5%). The branching coefficient *k*_b_ remained essentially unchanged, indicating that ARP2/3 amounts are not rate limiting for ARP2/3 mediated branching inside cortical condensates. The dominant effect of mild and moderate *arx-2* RNAi is an approximately 1.7- and 2.7-fold increase, respectively, of the F-actin loss rate *k*_d_. This is consistent with previous findings that the ARP2/3 complex protects F-actin from depolymerization in vitro^46^. In addition to the changes of coefficients, mild and moderate *arx-2* RNAi both reduced the average F-actin concentration by a factor of approximately 1.4 and 1.5, respectively, and increased the average WSP-1 concentration by a factor of approximately 1.5 and 1.8, respectively (Fig. 3d). In all three cases, no RNAi control, and mild and moderate *arx-2* RNAi, the pairs of average concentrations fall on the line of constant total density of WSP-1 and F-actin together (Fig. 3d, yellow dashed line; see Supplementary Information). We conclude that the ARP2/3 complex, largely through its impact on F-actin disassembly, governs the transition from condensate growth to condensate disassembly and determines the ensemble-averaged pair of internal concentration along the line of constant total density.

**Extended Data Fig. 8:**
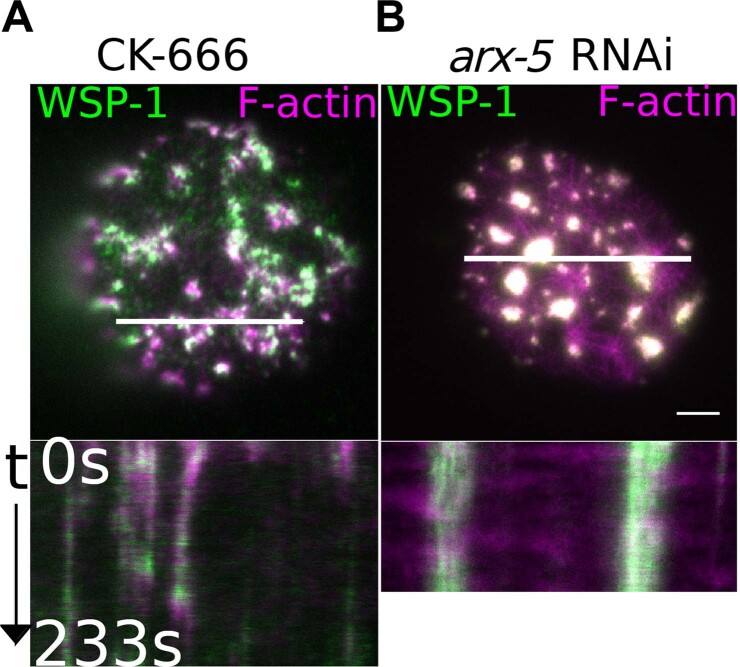
Alternative perturbations of the ARP2/3 complex. **A**,**B**, Snapshots (top) and kymographs (bottom) of *C. elegans* oocytes subjected to (**A**) CK666 drug treatment and (**B**) 24 h arx-5 RNAi (right). In both cases, as with 24 h of *arx-2* RNAi (Fig. 3b right), large persistent patches of WSP-1 and F-actin are observed. Scale bar, 10µm.

**Extended Data Fig. 9:**
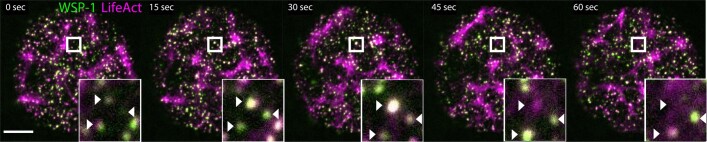
Representative example of local environment-independent condensate growth dynamics. A representative *C. elegans* oocyte cortex over time with zoomed insets showing three condensates (white arrowheads) sharing the same local environment but exhibiting compositionally distinct instantaneous growth. Another example is shown in Fig. 1d. Scale bar, 10µm.

Strong depletion of ARX-2 by RNAi (more than 20 h of RNAi feeding at 20 °C) resulted in a loss of dynamic cortical condensates, and a considerably altered cortical architecture with large persistent patches of F-actin and WSP-1 (Fig. 3b, right, Supplementary Video 5 and Extended Data Fig. 8). We asked if this phenotype can be understood given the condensate growth laws above. It is not possible to determine the four growth law coefficients for strong *arx-2* RNAi using mass balance imaging, because there are no dynamic condensates. However, the systematic change of both the F-actin loss rate *k*_d_ and the condensate number per oocyte for increasing strength of *arx-2* RNAi enabled us to provide a lower-bound estimate of *k*_d_ for the strong RNAi condition (Extended Data Fig. 7). We find that at and above this estimated value of *k*_d_ the system crosses a critical point at which the two nullclines switch their position, with the F-actin nullcline now above the WSP-1 nullcline (Fig. 3c, right). This causes a notable change in the growth dynamics of cortical condensates, with a complete loss of the homoclinic orbits that transition from growth to shrinkage. Instead, condensates exhibit unbounded growth consistent with the emergence of large persistent patches of F-actin and WSP-1 (Fig. 3b, right; note that we expect unbounded growth to ultimately become limited by effects we have not considered in our description, such as the depletion of the monomer pool). In conclusion, our analysis suggests that a switching of nullcline positions in strong *arx-2* RNAi leads to uncontrolled F-actin growth and impaired cortical activation in the oocyte, and therefore impaired later development^22,47^ (Supplementary Notes 5 and 6).

## Ensemble properties

**Fig. 4:**
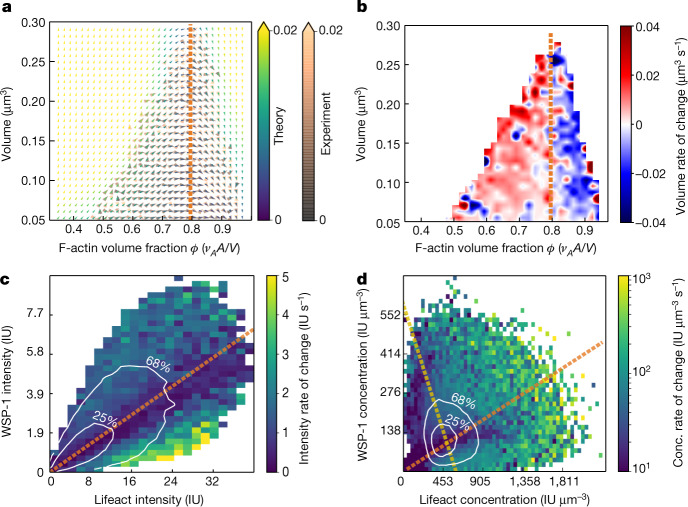
Ensemble average concentrations are determined by the stoichiometry with slowest dynamics. **a**, Phase portrait of condensate dynamics in the $\varphi$–$V$ plane. Orange and grey arrows are determined from measured WSP-1 and F-actin amounts and, in contrast to Fig. 2c, using measured condensate volumes based on the assumption that cortical condensates have a spherical shape. Yellow, green and blue arrows are calculated from the empirically determined growth laws expressed in the $\varphi$–$V$ plane. Colours denote time rate change vector magnitudes. The thick line denotes the experimentally determined volume nullcline. **b**, Rate of change of volume as a function of instantaneous volume and effective F-actin volume fraction. Condensates transition from growth to shrinkage at an effective F-actin volume fraction of 0.8 (the orange dashed line), which corresponds to the region of slow kinetics within the transition region (Figs. 2d and **c** and **d**). **c**, Mass flux phase portrait current magnitude. Contour lines (68%, 25%) depict the most commonly occupied total intensity values. The orange dashed line indicates the effective F-actin volume fraction corresponding to the centre of the transition region (Fig. 2d), and coincides with lowest total currents and slowest kinetics. **d**, Concentration flux phase portrait current magnitude. Contour lines (68%, 25%) depict the most commonly occupied concentration values and reflect the preferential maintenance of a pair of concentrations. This pair of concentrations lies at the intersection of the line of constant total density (yellow) and the line of dominant stoichiometry (orange), which corresponds to the orange lines in **a**–**c** and to the transition regions of Fig. 2c,d (see also Extended Data Fig. 5 for moderate *arx-2* RNAi conditions). Source data

How do the growth kinetics lead to a specific pair of average internal concentrations and therefore a specific stoichiometry? To address this question, we change variables from F-actin amount *A* and WSP-1 amount *W* to effective F-actin volume fraction $\varphi ={v}_{A}A/V$ and condensate volume *V*. Figure 4a shows that the calculated phase portrait in the $\varphi$–$V$ plane obtained by a change of variables of the empirically determined growth laws is consisted with the experimental one determined from measured WSP-1 and F-actin amounts and measured condensate volumes. Figure 4b shows that the transition from condensate growth (red) to shrinkage (blue) occurs at an effective F-actin volume fraction of approximately 0.8, corresponding to a stoichiometry of approximately 0.86. Notably, at this stoichiometry the rate of change of condensate volumes and stoichiometry is slowest (orange dotted lines in Fig. 4a,b,d), implying that the ensemble of dynamic condensates is governed by this slowly varying, thus dominant, stoichiometry. Hence, the peak of the concentration histograms (Figs. 1j and 3d) occurs at the point at which the line of dominant stoichiometry intersects with the line of constant total density (Fig. 4d).

## Intensive chemical reaction dynamics

We also recognized that the time evolutions of the effective F-actin volume fraction $\dot{\varphi }$ and the WSP-1 and F-actin concentrations are independent of condensate volume (Supplementary Notes 8–10). Thus, condensate dynamics are intensive, which is consistent with mass action kinetics in well-mixed systems. However, conventional mass action kinetics change reactant concentrations at constant volume, but usually do not involve assembly and disassembly as is the case here. Note that intensive condensate dynamics are not consistent with the conventional kinetics of nucleation and growth of liquid-like condensates, in which assembly rates depend on condensate size^48,49^. This reveals that cortical condensates exhibit an unconventional chemical kinetics in which mass action governs assembly and disassembly, and therefore the effect of mass action dynamics on concentrations in condensates is modified (Supplementary Notes 8–10). Note, however, that even though cortical condensates do not assemble via classical nucleation and growth, the intensive condensate dynamics show that the condensate material behaves as a well-mixed phase with size-independent properties. Intensive reaction dynamics are expected to arise in situations in which the time for diffusion across the condensate is shorter than the typical time associated with a chemical reaction. The condensate dynamic instability limits cortical condensate size. Therefore, reaction dynamics remain intensive and the resultant chemically active micro-emulsion maintains a steady-state size distribution (Fig. 1k)^11^.

## Discussion

To conclude, cortical condensates represent a new type of non-equilibrium biomolecular condensate that assembles and disassembles via a non-linear dynamic process governed by mass action chemical kinetics. They recruit molecules that drive branched nucleation of F-actin and support the activation of the actomyosin cortex. The dynamics of the growth and shrinkage of cortical condensates are similar to the dynamic instability of growing and shrinking microtubules^10^, but arises in a bulk assembly that forms a phase. We suggest that the formation and subsequent dissolution of cortical condensates via a condensate dynamic instability serves to control autocatalytic F-actin nucleation and prevents runaway growth during the activation of the first cortical actin meshwork in the *C. elegans* oocyte.

## Methods

See Supplementary Methods.

### Reporting summary

Further information on research design is available in the Nature Research Reporting Summary linked to this article.

## Online content

Any methods, additional references, Nature Research reporting summaries, source data, extended data, supplementary information, acknowledgements, peer review information; details of author contributions and competing interests; and statements of data and code availability are available at 10.1038/s41586-022-05084-3.

## Supplementary information

Supplementary Information Supplementary Methods, Notes 1–10 and references.

Reporting Summary

Supplementary Table 1 Transgenic strains used in this study: of the listed strains, GFP::Utrophin and PH-domain::gfp were generated by knock-in transgenesis, LifeAct::mKate was generated with Mos1-mediated Single-Copy Insertion (MosSCI), and all other fluorescent labelling of proteins are expressed from the endogenous locus with CRISPR mutagenesis, from the work of A. C. Reymann and a generous gift from the Guangxu Ou laboratory.

Supplementary Video 1 In utero confocal spinning disc imaging of *C. elegans* oocyte to embryo transition. LifeAct::mKate labellng F-actin in magenta and histones H2B::GFP labelled in green. Scale bar, 10 µm. Time stamp (min:s).

Supplementary Video 2 Formation of an actomyosin cortex in an isolated C. *elegans* oocyte. LifeAct::mKate labelling F-actin in magenta and non-muscle myosin NMY-2::GFP labelled in green. Scale bar, 10 µm. Time stamp (min:s).

Supplementary Video 3 Cortical condensates in a *C. elegans* oocyte undergoing actomyosin cortex formation. LifeAct::mKate labelling F-actin in magenta and endogenously labelled WSP-1::GFP in green. Scale bar, 10 µm. Time stamp (min:s).

Supplementary Video 4 Cortical condensates in a *C. elegans* oocyte contain WSP-1 and ARX-2. Endogenously labelled WSP-1::GFP in green and endogenously labelled ARX-2::mCherry in blue. Scale bar, 10 µm. Time stamp (min:s).

Supplementary Video 5 Oocyte strongly depleted of ARX-2 after 24 h of RNAi feeding. LifeAct::mKate labelling F-actin in magenta and WSP-1::GFP labelled in green. Scale bar, 10 µm. Time stamp (min:s).

Supplementary Video 6 Oocyte moderately depleted of ARX-2 after 19 h of RNAi feeding. LifeAct::mKate labelling F-actin in magenta and endogenous WSP-1::GFP labelled in green. Scale bar, 10 µm. Time stamp (min:s).

Supplementary video 7 SIM-TIRF movie of *C. elegans* oocyte. LifeAct::mKate labelling F-actin in red and non-muscle myosin NMY-2::GFP labelled in green. Scale bar, 10 µm. Time stamp (min:s).

## Figures and Tables

**Extended Data Fig. 2:**
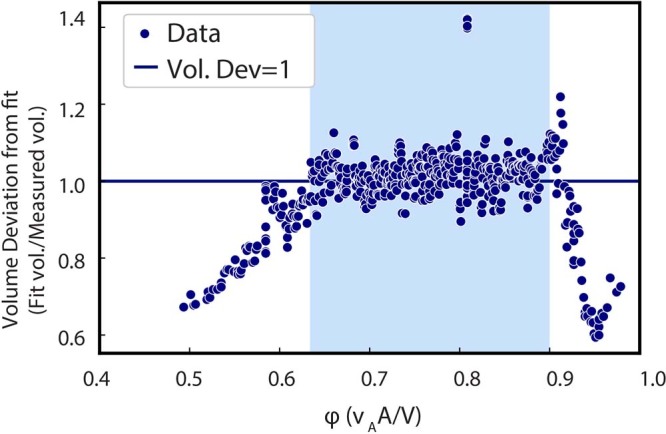
Volume dependence on molecular content. Deviation of measured volume from linear combination of WSP-1 and F-actin intensities for F-actin volume fractions φ <~ 0.6 and φ >~ 0.9. Note the deviation from linearity in Fig. 2e,f coincides with the range of volume fractions where measured volume deviates from V = v_A_A + v_W_W. Source data

**Extended Data Fig. 5:**
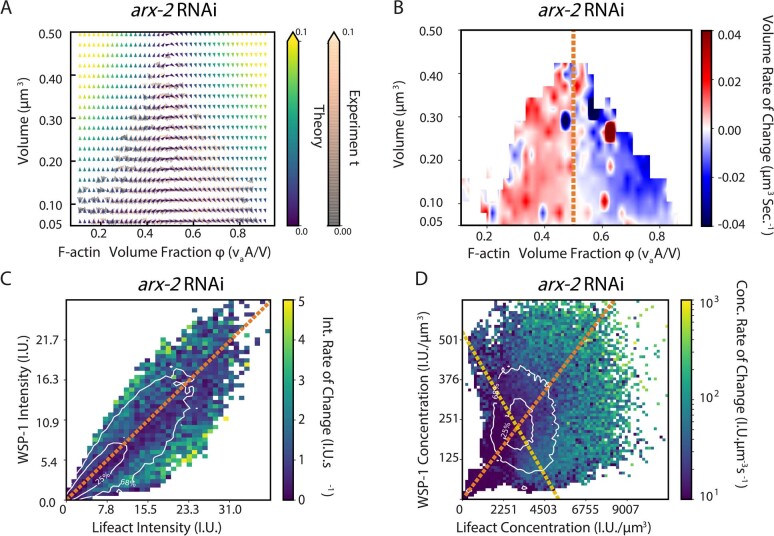
Volume-independent stoichiometry evolution sets internal concentrations also for moderate arx-2 RNAi. **A**, Volume vs. F-actin volume fraction phase portrait obtained by a change of variables of the moderate *arx-2* RNAi data represented in Fig. 3. Orange-grey arrows, measured as in Fig. 2c. Yellow-green-blue, calculated using the empirically determined growth laws. Colors denote time rate change vector magnitudes. The time evolution of effective F-actin volume fraction is independent of volume (also see supplement). **B**, Rate of change of volume (blue-red heatmap) as a function of instantaneous volume and effective F-actin volume fraction. Condensates transition from growth to shrinkage at an effective F-actin volume fraction of 0.5 (orange-dashed line), which corresponds to the region of slow kinetics within the transition region (Fig. 2d, Fig. 4c,d). **C**, Mass flux phase portrait current magnitude. Contour lines (68%, 25%) depict the most commonly occupied total intensity values. The orange dashed line indicates the effective F-actin volume fraction corresponding to the center of the transition region (Fig. 2d), and coincides with lowest currents and slowest kinetics. **D**, Concentration flux phase portrait current magnitude. Contour lines (68%, 25%) depict the most commonly occupied concentration values and reflect the preferential maintenance of a pair of concentrations. This pair of concentrations lies at the intersect of the line of constant total density (yellow) and the line of dominant stoichiometry (orange) which corresponds to the orange lines in (**A**-**C**) and to the transition regions of Fig. 2d. Source data

**Extended Data Fig. 10:**
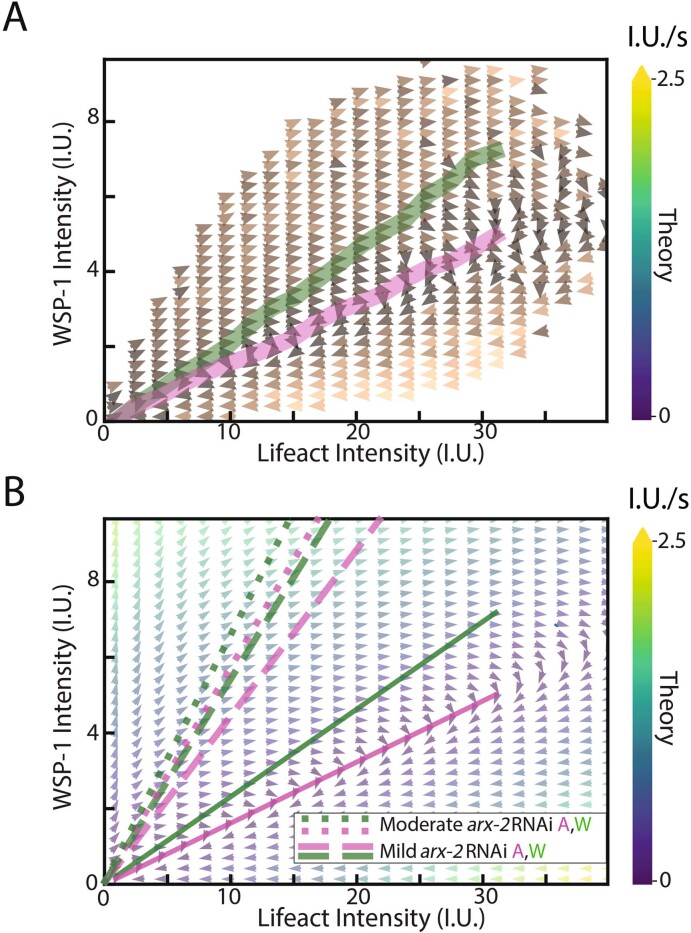
Theory and experimental phase portraits from Fig. 2c presented separately. **A**, Non-RNAi experimental phase portrait from Fig. 2c. **B**, Non-RNAi theory phase portrait from Fig. 2c. Solid lines indicate WSP-1 (green) and F-actin (Magenta) nullclines, dashed lines indicate WSP-1 (green) and F-actin (magenta) mild *arx-2* RNAi nullclines, and dotted lines indicate WSP-1 (green) and F-actin (magenta) moderate *arx-2* RNAi nullclines.

## Data Availability

Condensate track data are publicly available at 10.17617/3.PIRFA2. Source data are provided with this paper.

## Source data

Source Data Fig. 1
Source Data Fig. 2
Source Data Fig. 3
Source Data Fig. 4
Source Data Extended Data Fig. 2
Source Data Extended Data Fig. 3
Source Data Extended Data Fig. 5
Source Data Extended Data Fig. 7

## Code availability

Custom code publicly available at 10.17617/3.PIRFA2. 
