# Publisher Correction: A condensate dynamic instability orchestrates actomyosin cortex activation

**DOI:** 10.1038/s41586-023-05932-w

**Published:** 2023-03-16

**Authors:** Victoria Tianjing Yan, Arjun Narayanan, Tina Wiegand, Frank Jülicher, Stephan W. Grill

**Affiliations:** 1 Max Planck Institute of Molecular Cell Biology and Genetics (MPI-CBG), Dresden, Germany; 2 Biotechnology Center, TU Dresden, Dresden, Germany; 3 Max Planck Institute for the Physics of Complex Systems (MPI-PKS), Dresden, Germany; 4 Center for Systems Biology Dresden (CSBD), Dresden, Germany; 5 Cluster of Excellence Physics of Life, TU Dresden, Dresden, Germany

**Keywords:** Intrinsically disordered proteins, Germline development

Correction to: *Nature* 10.1038/s41586-022-05084-3 Published online 17 August 2022

In the version of this article initially published, in the legend for Fig. 3c, WSP-1 was erroneously denoted as magenta and F-actin as green. The corrected text now reads “Thick lines indicate the measured WSP-1 (green) and F-actin (magenta) nullclines”. In addition, in the x-axis label of Fig. 4d, “Lifeact” erroneously appeared as “Lifetime”. The corrected label now reads “Lifeact concentration (IU μm^–3^)”. The errors have been corrected in the HTML and PDF versions of the article.

