# Supplementary material for: A condensate dynamic instability orchestrates actomyosin cortex activation
Source: Nature. 2022 Aug 17;609(7927):597–604. doi: 10.1038/s41586-022-05084-3 (PMC9477739; doi:10.1038/s41586-022-05084-3)
Supplement: Supplementary file 1 — Supplementary Methods, Notes 1–10 and references. [file 41586_2022_5084_MOESM1_ESM.pdf]

nature portfolio

https://doi.org/10.1038/s41586-022-05084-3

Supplementary information

A condensate dynamic instability orchestrates actomyosin cortex activation

In the format provided by the authors and unedited

Supplementary Materials for

A condensate dynamic instability orchestrates acto-myosin cortex activation

Victoria Tianjing Yan[1,2,†], Arjun Narayanan[1,2,3,4,†,*], Tina Wiegand[1,3,4], Frank Jülicher[3,4,5,*], Stephan W. Grill [1,4,5,*]

[1]*Max Planck Institute of Molecular Cell Biology and Genetics (MPI-CBG), Dresden, Germany*

[2]*Biotechnology Center, TU Dresden, Dresden, Germany*

[3]*Max Planck Institute for the Physics of Complex Systems (MPI-PKS), Dresden, Germany*

[4]*Center for Systems Biology Dresden (CSBD), Dresden, Germany*

[5]*Cluster of Excellence Physics of Life, TU Dresden, Dresden, Germany*

† These authors contributed equally.

* Correspondence: Correspondence and requests for materials should be addressed to:

A.N. (email: anarayan@mpi-cbg.de), F.J. (email: julicher@pks.mpg.de),

S.W.G. (email: grill@mpi-cbg.de).

Supplementary Methods

C. elegans **maintenance and strains** The *C. elegans* strains used in this study were cultured on nematode growth media plates, seeded with OP50 *E. coli*. Strains were incubated in 20 °C until imaging at 22-24 °C. The transgenic strains used in this study are listed in Supplementary Table 1.

***In utero* confocal imaging** Adult *C. elegans* hermaphrodites were anaesthetized for 3 minutes with 0.5% Tetramisole (Sigma-Aldrich T1512) in M9 buffer, then placed on an 18x18 mm high-precision coverslip precoated with 0.1% Poly-L-Lysine (Sigma-Aldrich P8920). Images were obtained on a spinning disk confocal microscope (Zeiss C-Apochromat, 63x/1.2 NA objective, with Hamamatsu Orca-Flash 4.0 camera). Images were acquired at 3 second intervals with 3 Z-slices (totalling 1.5 μ m). The excitation lasers used were 488 and 568 nm, and the exposure time was 100ms for each channel.

Intensity units Intensity units (I.U.) for both the F-actin and WSP-1 channels correspond to the camera readings in integrated condensate total intensity divided by 10,000 for ease of presentation and as a proxy for molecular amounts. Absolute molecular numbers can be estimated for WSP-1 but not for F-actin because of our labeling strategy - for WSP-1 condensates we estimate condensates contain a few hundred WSP-1 molecules.

Oocyte TIRF imaging Oocytes of adult hermaphrodites were dissected as described in reference[1], and imaged in Shelton's growth media[2]. 1% 20 μm polystyrene beads were used in the growth media to act as a spacer for imaging. Cells imaged in TIRF microscopy were placed on ethanol cleaned 18x18 mm high-precision coverslips. We pursued TIRF imaging under HILO TIRF conditions[3] to enable imaging and quantification of objects that extend beyond the depth of the evanescent TIRF field. HILO TIRF microscopy of oocytes was conducted on a Nikon Ti microscope equipped with a TIRF turret. The alignment and collimation of TIRF lasers were performed at the beginning of each imaging session. Movies were acquired at 1s intervals, with 100ms ex-

posure time for each channel. The laser lines used were 488nm, and 568nm lasers, for GFP, mKate/mCherry, and JF647 respectively. The images were acquired on an Andor iXon-Ultra EM-CCD with 1.5x optical zoom. Nikon Perfect Focus was used to maintain focus of the TIRF plane, throughout the duration of imaging. The angle of incidence for HILO TIRF imaging[3] was set at approximately 2° below the critical angle for TIRF imaging. Imaging and subsequent image analysis was tested using flourescent microscopheres of 0.1 to $4\mu m$ diameter (data not shown). A 488 nm laser was used with a 525/50 bandpass emission filter and a 561 nm laser was used with a 568 nm long pass filter, for imaging GFP and mKate respectively. Oocyte extraction from the *C. elegans* gonad did not influence condensate lifetime (Movie S1).

Kymographs Kymographs were used to illustrate dynamics in time. Line kymographs were generated in Fiji/ImageJ.

Oocyte chemical inhibitor treatments Chemical inhibitors were directly added to Shelton's growth media during the dissection stage, as they are cell-permeable, and the oocytes have no eggshell. CK-666 was used at 10 μM.

RNAi Feeding Knockdowns of proteins were achieved by RNAi clone feeding to L4 stage *C. elegans*. RNAi clones were acquired from the Ahringer lab RNAi library, and seeded onto a feeding plate (NGM agar containing 0.5 mM to 2 mM IPTG and 25μg/ml carbenicillin). L4 stage worms were placed onto feeding plates and incubated at 20°C for 15 to 30 hours, depending on the intended strength of depletion.

Statistics and Reproducibility The representative images in the main text and extended data figures correspond to multiple replicates as follows: Figure 1A - 12 independent experiments; Figure 1B - 9 independent experiments; Figure 1C - 10 independent experiments; Figure 3A - 9 independent experiments; Figure 3B - 7,7,9 independent experiments from left to right; extended data figure 1A - 12 independent experiments; extended data figure 1B - 4 independent experiments; extended data figure 1D - 4 independent experiments; extended data figure 1E - 3 independent experiments.

Phase portrait analysis pipeline Microscopy images used for analysis were first exported using Fiji/ImageJ. Background subtraction of the camera noise is performed with a custom Matlab script. Intensities from both GFP and mKate channels were summed prior to segmentation, in order to capture full duration of tracks. Puncta were segmented with Ilastik[4] using version 1.4.0b14 and the *simple segmentation* method [5]. Intensities of segmented puncta were measured using Matlab regionprops. Tracking of the puncta traces were performed with Fiji Trackmate (Simple LAP tracker)[6]. Condensate volumes V were calculated from the segmented area A assuming a spherical shape as $V = \frac{4A^{3/2}}{3\pi^{1/2}}$. Track intensity parsing, as well as phase portrait generation from data, and nullcline calculation were performed with Matlab and Python custom written code. Scripts and raw track intensity data are available at Edmond – The Open Research Data Repository of the Max Planck Society[7].

Fitting procedure for estimating parameters Least squares fitting of a straight line to the $\frac{\dot{W}}{W}$ vs $v_A A/(A + W)$ data was performed. The slope of this straight line corresponds to k_l, while the

offset corresponds to k_r. Similarly fitting for performed for the F-actin growth rate(Figure 2 E,F). Volume coefficients v_A and v_W were obtained estimating the volume of clusters in the microscopy images for clusters larger than the lateral resolution $\sim$ 200 nm. Fitting was done using the 2D 'leastsq' method of Python Lmfit. The values of the coefficients estimated by this fitting procedure are:

For the No-RNAi dataset (Figure 2C): $k_d = 0.15(\pm 0.02)$ and $k_r = 0.54(\pm 0.02)$, $k_l/v_A = 0.73(\pm 0.03)$ and $k_b/v_W = 0.78(\pm 0.02)$. Volume $V = v_A A + v_W W$ with $v_A = 1.54 \cdot 10^{-7}(\pm 1 \cdot 10^{-8}) \mu m^3/I.U.$ and $v_W = 2.34 \cdot 10^{-7}(\pm 2 \cdot 10^{-8}) \mu m^3/I.U.$

For Mild ARX-2 RNAi dataset (Figure 3C, left): $k_d = 0.25(\pm 0.02)$ and $k_r = 0.42(\pm 0.02)$, $k_l/v_A = 0.74(\pm 0.04)$ and $k_b/v_W = 0.65(\pm 0.04)$. Volume $V = v_A A + v_W W$ with $v_A = 2 \cdot 10^{-7}(\pm 2 \cdot 10^{-8}) \mu m^3/I.U.$ and $v_W = 2.7 \cdot 10^{-7}(\pm 2 \cdot 10^{-8}) \mu m^3/I.U.$.

For Moderate ARX-2 RNAi dataset (Figure 3C, middle): $k_d = 0.39(\pm 0.02)$ and $k_r = 0.45(\pm 0.02)$, $k_l/v_A = 0.94(\pm 0.04)$ and $k_b/v_W = 0.81(\pm 0.04)$. Volume $V = v_A A + v_W W$ with $v_A = 2.3 \cdot 10^{-7}(\pm 2 \cdot 10^{-8}) \mu m^3/I.U.$ and $v_W = 3.7 \cdot 10^{-7}(\pm 2 \cdot 10^{-8}) \mu m^3/I.U.$.

For the strong RNAi predicted phase portrait of Figure 3, the moderate ARX-2 RNAi values were used but with $k_d = 0.51$ just above the estimate shown in Figure S7 B . Mild and Moderate ARX-2 RNAi datasets corresponded to $\sim$ 18 to 20 hours and to $\sim$ 19 to 20 hours of ARX-2 RNAi, respectively, and we distinguished between the severity of phenotype via the different numbers of condensates in steady state (Figure S7 B, left). Across the no-RNAi, Mild ARX-2 RNAi and

Moderate ARX-2 RNAi, the major change in coefficients is in k_d and the other changes while significant from a statistical point of view are not major.

Supplementary note 1: Dependence of condensate volume on F-actin and WSP-1 amounts

In the main text we showed that the volume of a cortical condensate can be accounted for by a linear combination of F-actin and WSP-1 amounts (Figure 1 I). Note that condensate volumes V were calculated from the segmented area A in our microscopy images assuming a spherical shape (see above). Approximately 80% of condensates volume measurements are in a range of 0.6 to 0.9 of F-actin volume fraction $\frac{v_A A}{v_A A + v_W W}$ (corresponding to a range of ~ 0.65 to ~ 0.93 of stoichiometry $\frac{A}{A+W}$). For all events in this range, the measured volume can be described by $V = v_A A + v_W W$ (Figure S2). All stoichiometries displayed in Figures 2-4 lie in this range.

We note that the effective volume coefficients v_A and v_W are the condensate volume per WSP-1 or F-actin intensity unit. The observation that the volume can be accounted for by a sum of F-Actin and WSP-1 volume contributions indicates that additional components enter the condensate at fixed stoichiometries with respect to WSP-1 or F-actin. Therefore the volume coefficients do not only contain the molecular volumes of WSP-1 and F-Actin, but those of additional molecules that are recruited . Note that under ARX-2 RNAi experiments these coefficients change, This is reasonable, because the RNAi conditions can modulate both internal structure and the stoichiometries at which additional components are recruited to the condensate.

Supplementary note 2: Condensate stoichiometry and effective F-actin volume fraction

In the main text, based on measured intensities alone, we define stoichiometry S= $\frac{A}{A+W}$. This also corresponds to a compositional fraction of F-actin. Based on both measured intensities and the measured volumes, we define the effective F-actin volume fraction $\phi = \frac{v_A A}{v_A A + v_W W}$. Given our estimation of the volume contribution coefficients v_A and v_w (see Section 2 and Figure 1 I), the two definitions are related by $\phi = \frac{S}{S - \frac{v_W}{v_A}(1-S)}$. Thus the threshold intensity stoichiometry of $\sim .86$ (between the nullclines at $S = 0.85$ and $S = 0.89$ in Figure 2) corresponds to F-actin volume fraction of $\phi = 0.8$ (as seen in the orange line in Figure 4).

Supplementary note 3: Determination of empirical condensate growth laws

The experimental data enforces constraints on the dependencies of F-actin and WSP-1 growth rate on internal composition (A and W amounts). These constraints are simultaneously captured in the linear dependence of the measured relative growth rates ($\frac{\dot{A}}{A}$, $\frac{\dot{W}}{W}$) as a function of the effective F-actin volume fraction ($\phi = v_A A/V$) (main text Figure 2 E,F). The positive intercept in the case of $\frac{\dot{W}}{W}$ as a function of ϕ fixes both the existence and form of the WSP-1 self recruitment term ($+k_r W$) while the negative intercept in the case of $\frac{\dot{A}}{A}$ as a function of $1-\phi$ fixes the existence and form of the F-actin loss term ($-k_d A$). Additionally, the fact that the relative growth rates for F-actin and WSP-1 change linearly with F-actin volume fraction ϕ (or $1-\phi$) fixes the existence and form of the cross terms ($+k_b \frac{AW}{V}$ and $-k_l \frac{AW}{V}$). Thus, the experimentally observed linearity of the functions $\dot{W}/W = k_r - \frac{k_f}{v_A}\phi$ and $\dot{A}/A = \frac{k_l}{v_W}(1-\phi) - k_d$ with respect to ϕ completely determines the form of

all four terms in the growth laws. Together these growth laws show continuous compositional and size evolution throughout the lifetime of condensates. This behaviour reveals the non-equilibrium nature of condensates. The dynamic instability highlights the nonlinear dynamical behaviour. In this sense, condensates are *far-from-equilibrium* as we state in the main text.

This mathematical form is also reflected in the phase portrait slices of Figure S3. The dependencies of $\dot{W}$ and $\dot{A}$ on A and W include contributions from two terms each : $k_r W$ and $-k_l \frac{AW}{V}$ in the case of $\dot{W}(A, W)$ and $-k_d A$ and $+k_b \frac{AW}{V}$ in the case of $\dot{A}(A, W)$. Figure S3 D shows that at low WSP-1 levels, F-actin is lost with a rate that grows linearly with A ($-k_d A$). At the low WSP-1 levels of Figure S3 D the contribution from the $+k_b \frac{AW}{V}$ term is minimal allowing the linear dependence to be directly visible. Similarly the form of the cross dependencies ($-k_l \frac{AW}{V}$ and $+k_b \frac{AW}{V}$) are reflected in the linear-to-saturating behavior of the Figure S3 B,C just as the functions $-k_l \frac{AW}{V}$ and $+k_b \frac{AW}{V}$ should behave with $V = v_A A + v_W W$. Finally the non monotonic behaviour of Figure S3 A reflects the competing contributions of both WSP-1 self recruitment and F-actin dependent WSP-1 loss. Importantly, these dependencies recapitulate the full phase portraits in both control (Figure 2 C, Figure 4 A) and ARX-2 RNAi (Figure 3 C, Figure S5) conditions. Note that the growth laws in both control and two distinct ARX-2 RNAi conditions simultaneously recapitulate phase portraits resulting from two distinct measurements in two distinct coordinate systems - intensity measurements for the mass flux phase portrait of Figure 2 C (3C for mild ARX-2 RNAi, S7 for moderate ARX-2 RNAi) and volume measurements for the volume-volume fraction phase portrait of Figure 4 A (S5 for moderate ARX-2 RNAi).

Supplementary note 4: RNAi perturbations

In Figure S4, we provide representative images, kymographs and quantifications detailing the phenotypes that result from RNAi perturbations. Figure S4 A shows the effect of 24hr RNAi against ARX-2 - this lowers the ARX-2 levels at the cortex (via fluorescence, left 2 panels) and simultaneously leads to a disappearance of dynamic condensates and an appearance of large stable WSP-1 and F-actin patches (Right 2 panels). Similar results are obtained on other distinct but interdependent [8] perturbations of the ARP2/3 complex (Figure S8). As discussed in the main text and the next supplementary note the large stable patches of F-actin and WSP-1 are linked to a crossing of nullclines on progressive ARX-2 depletion. Across ARX-2 RNAi perturbations of Figure 3 and Figure S4, cytoplasmic WSP-1 levels are unchanged. Figure S4 B-D show the effect of 24hr RNAi against UNC60, CAP-1 and COR-1 respectively - In the case of UNC60 and CAP-1 we directly measure the effect of RNAi on UNC60 and CAP-1 levels (via fluorescence, B,C left two panels) while in the case of COR-1 we quantify the effect of RNAi by measuring an increase in the F-actin levels at the cortex (D, left two panels). In all three cases, large stable WSP-1 and F-actin accumulations result and dynamic condensates are abolished. Our growth laws establish that ARX-2 plays a role in F-actin stabilizing/destabilization by suppressing the F-actin loss rate coefficient (k_d). UNC60, CAP-1 and COR-1 are also expected to affect the stability and growth of F-actin filaments. Taken together, these four RNAi conditions (A-D) suggest that condensate dynamics are strongly affected by modulation of F-actin filament stability and growth. Conversely, Figure S4 E-I show the persistence of dynamic cortical condensates with RNAi against RHO-1, CYK-1 CDC42, CHIN-1 and in a constitutively inactive NCK-1 mutant[9] strain respectively. In the case

of RHO-1 and CYK-1 we measure the efficacy of RNAi by measuring the RHO-1 and CYK-1 levels at the cortex respectively (via flourescence, S4 E,F left two panels) in the case of the WSP-1 activity regulators CDC42 and CHIN-1 we measure the efficacy of RNAI by measuring the change in cortical WSP-1 levels (via fluorescence S4 G,H left two panels). In all 5 cases (E-I, right panels) dynamic condensates persist. Taken together we find that the nucleation and growth of dynamic cortical condensates is robust to variations ($\sim$ 50 percent, see Figure S4) in the levels of both the RHO-1/Formin pathway and the CDC42/WSP-1 pathway and to NCK-1 activity but not robust to perturbations of F-actin filament stability and growth.

Supplementary note 5: Transition from bounded to unbounded growth

According to the growth laws, for values of the F-actin loss coefficient k_d smaller than ~ 0.52 (with the other parameters determined for moderate ARX-2 RNAi) condensates, after nucleation, follow homoclinic orbits that first grow mainly in the W direction before reaching a maximal size prior to returning towards (0,0) mainly along the A-direction. These dynamics arise because the W-nullcline lies above the A-nullcline. For values of k_d that are larger than~ 0.52 a drastically different behaviour is observed: condensates, after nucleation, exhibit unbounded growth. The dynamics are different because the two nullclines switch their position, with the A-nullcline now above the W-nullcline.

In Figure 2,3 we perform mass balance imaging of dynamic cortical condensates for 3 progressive levels of ARX-2 (no-RNAi control, mild and moderate ARX-2 RNAi). We show that

the W and A nullcines move progressively closer to each other with increasing severity of ARX-2 RNAi, while also the number of dynamic cortical condensates per oocyte decreases (Figure S7A). In Figure S7B we show that the F-actin loss coefficient k_d increases with increasing severity of ARX-2 RNAi. Since we expect no dynamic condensates when the nullclines switch position, the critical value of kd at which the two nullclines switch position should correspond to the case of zero dynamic condensates. Indeed, the value of k_d estimated from this graph for zero dynamic condensates condensates (~ 0.53) is close but slightly larger than the critical value of k_d at which the A and W nullclines switch position in our theoretical analysis (see paragraph above). Thus, our estimates from three progressively strong RNAi conditions predict a transition to unbounded growth, and the expected formation of large persistent patches of F-acting and WSP-1 as is observed for strong ARX-2 RNAi.

Supplementary note 6: Comparing dynamic cortical condensates with the microtubule dynamic instability

Microtubules are well known to exhibit a dynamic instability which switches them stochastically between a growing state and a shrinking state via a catastrophe event. Occasionally, shrinking microtubules can recover, and switch to the growing state via a rescue event [10]. Considering a collection of microtubules, these dynamics can give rise to a regime of bounded growth where the average length of microtubules remains finite, and a regime of unbounded growth where the average length increases as long as tubulin heterodimers are avaliable [11].

This behaviour can be compared to the dynamics of cortical condensates in our experiments, which we consider to exhibit dynamics that shares key features with the dynamic instability of microtubules. A single condensate follows a similar trajectory as a single microtubule. It is first nucleated, exhibits rapid growth before transitioning to shrinkage which can lead to complete disassembly. While microtubules grow as linear objects, cortical condensates grow in volume and in the three dimensions. Because of the transition from growth to shrinkage that is similar to microtubules, cortical condensates can also exhibit regimes of bounded and unbounded growth (Figure 3C). While the transition between growth and shrinkage in microtubules is governed by the GTP cap, here the transition is governed by the underlying biochemical reactions that increase stoichiometry with time and cause it to cross a threshold stoichiometry for shrinkage. Similar to microtubules, cortical condensates can also occasionally show rescue events (Figure S7C).

Supplementary note 7: Nucleation and growth of cortical condensates

Note that the condensate growth laws describe the dynamics of nucleated cortical condensates, but not the nucleation process. Nucleation appears to be a stochastic and depends on ARX-2 and thus the nucleation/branching pathway (Figure 3, Figure S7A). CDC42 RNAi does not appear to affect the number of cortical condensates (Figure S4). Dynamic cortical condensates are observed at specific time point within the cell cycle. This suggests that cell cycle progression generates an environment where robust and frequent condensate nucleation occurs. We note also that just nucleated condensates do have non-zero WSP-1 and F-actin amounts as needed for subsequent growth.

In the regime where condensate growth is bounded and cortical condensates shrink after reaching a maximal size, the cortex then consists of a large number of dynamic condensates which nucleate, grow, and then disappear. Micro-emulsions are characterized by small droplets that are prevented from coarsening to larger droplets and a macroscopic phase. Here we identify a new mechanism that can maintain small condensate sizes via a dynamic instability in an active system. Thus, cortex activation proceeds through an intermediate state that can be viewed as an active micro-emulsion.

Supplementary note 8: Volume independent condensate dynamics

Intensive properties defining a phase are those properties that are independent of volume (eg. concentration). By intensive condensate dynamics we mean the volume-independent time-evolution of the intensive properties defining the condensate phase. In the main text we stated that the growth laws imply a volume independent time-evolution of F-actin volume fraction $\phi = v_A A/V$, WSP-1 concentration $C_W = W/V$ and F-actin concentration $C_A = A/V$. Below we show that the time evolution of C_W, C_A and ϕ depends only on internal concentrations and not on condensate size.

We start from the growth laws as extracted from the experimental data

$$\dot{W} = k_r W - k_l \frac{AW}{V} \tag{1}$$

$$\dot{A} = k_b \frac{AW}{V} - k_d A \quad , \tag{2}$$

with $V = v_A A + v_W W$, and k's as defined in main text. We first divide the equations by volume

V to get

$$\frac{\dot{W}}{V} = k_r C_W - k_l C_A C_W \tag{3}$$

$$\frac{\dot{A}}{V} = k_b C_A C_W - k_d C_A \quad , \tag{4}$$

where $C_A = A/V$ and $C_W = W/V$ are the internal concentrations. Using $V = v_A A + v_W W$ it follows that

$$\frac{\dot{V}}{V} = v_A(k_b C_A C_W - k_d C_A) + v_W(k_r C_W - k_l C_A C_W) \quad , \tag{5}$$

which is a function of concentrations. Concentrations evolve according to $\dot{C_W} = \dot{W}/V - C_W \dot{V}/V$ and $\dot{C_A} = \dot{A}/V - C_A \dot{V}/V$. We thus get

$$\dot{C_W} = k_r C_W - k_l C_A C_W - C_W \frac{\dot{V}}{V} \tag{6}$$

$$\dot{C_A} = k_b C_W C_A - k_d C_A - C_A \frac{\dot{V}}{V} \quad . \tag{7}$$

Thus, Using Eq. (5) reveals that $\dot{C_A}, \dot{C_W}$ are determined by concentrations alone and are independent of condensate volume. This reveals intensive reaction dynamics.

We can finally express the dynamics in terms of the effective F-actin volume fraction $\phi = v_A C_A$ with $1 - \phi = v_W C_W$, and the volume $V = v_A A + v_W W$.

Using Eq. (5-7) we obtain the time rate of change of V and ϕ as functions of volume and stoichiometry:

$$\dot{V} = V\left[k_r(1-\phi) - k_d\phi + (\frac{k_b}{v_W} - \frac{k_l}{v_A})\phi(1-\phi)\right] \tag{8}$$

$$\dot{\phi} = (\frac{k_b}{v_W} - k_d - k_r)\phi + (k_d + k_r - \frac{2k_b}{v_W} + \frac{k_l}{v_A})\phi^2 + (\frac{k_b}{v_W} - \frac{k_l}{v_A})\phi^3 \quad . \tag{9}$$

Thus the evolution of F-actin volume fraction is given by a polynomial in ϕ and is independent of volume.

Supplementary note 9: Mass action kinetics during assembly and disassembly of cortical condensates

The law of mass action is typically applied to chemical reactions in a well-mixed container of fixed volume and states that the rate of a chemical reaction is proportional to the product of the activities or concentrations of the reactants (see for instance reference [12]). For example, consider n_A molecules of type A and n_B molecules of type B, whose one-step reaction produces molecule C, within a container of constant volume V. In this case, changes in the number of product molecules n_C arise from the rate at which molecules of type A and molecules of type B meet and react. Since the solution is well-mixed, the number of type A molecules within reaction-range of type B molecules at any instant is just the probability that the random placement of n_A molecules of type A and n_B molecules of type B within a Volume V results in such an overlap. This probability is proportional to $n_A n_B/V$ (for dilute systems) and thus,

$$\dot{n_C} = k\frac{n_A n_B}{V} \quad , \tag{10}$$

where k is the kinetic coefficient for production of C from proximity of A to B. Since the volume of the reaction container is a constant, on dividing by this volume, we get the familiar expression for the law of mass action for concentration changes

$$\dot{C}_c = kC_A C_B \quad , \tag{11}$$

where C_c, C_A and C_B denote the concentrations n_C/V, n_A/V and n_B/V respectively and the resulting time-evolution of C_c is intensive - i.e. depending only on the intensive quantities A_c, B_c and independent of volume V.

In the case of cortical condensates, the volume is not constant as the reaction container itself evolves with molecular amounts. A key result of our work is that mass action kinetics describe cortical condensate evolution at any instantaneous volume. To see this, first consider the branching reaction with two reactants A and W at concentrations $C_A = A/V$ and $C_W = W/V$. The rate at which branching reactions lead to a loss of WSP-1 ($\dot{W} = -k_l AW/V$) or addition of F-actin ($\dot{A} = k_b AW/V$) are proportional to collision rates of the two reactants A and W in a well mixed container as they have the form of the product of concentrations $C_A C_W = AW/V^2$ summed over the instantaneous condensate volume V. Second, consider WSP-1 self-recruitment rate ($k_r W$), WSP-1 self recruitment can be considered a reaction between internal WSP-1 at the concentration C_W and a constant permeating bath of recruitable WSP-1. Then, this term has the form of the product of internal WSP-1 concentration with the constant bath concentration and summed over the instantaneous condensate volume V. Lastly, the F-actin loss rate ($-k_d A$) requires a single reactant (A) to depolymerize and is also proportional to concentrations of this reactant C_A summed over the instantaneous condensate volume.

Thus the growth laws:

$$\dot{W} = k_r W - k_l \frac{AW}{V} \tag{12}$$

$$\dot{A} = k_b \frac{AW}{V} - k_d A \quad , \tag{13}$$

are consistent with mass action kinetics in the instantaneous condensate volume V. Dividing by V, we get

$$\dot{W}/V = k_r C_W - k_l C_A C_W \tag{14}$$

$$\dot{A}/V = k_b C_A C_W - k_d C_A \quad . \tag{15}$$

However, as V varies with time, the left hand sides are not simply the rate of change of WSP-1 and F-actin concentrations $\dot{C_W}$, $\dot{C_A}$. Instead, since $\dot{C_W} = W/V$ and $\dot{C_A} = A/V$ we get

$$\dot{C_W} = k_r C_W - k_l C_A C_W - C_W \dot{V}/V \tag{16}$$

$$\dot{C_A} = k_b C_A C_W - k_d C_A - C_A \dot{V}/V \quad . \tag{17}$$

The additional terms $C_W \dot{V}/V$ and $C_A \dot{V}/V$ on the right hand side arise from volume changes. In order for the evolution of internal concentrations to be volume independent (intensive), $\dot{V}/V$ must be volume independent.

In the case of cortical condensates, the number of molecules evolve according to mass action rates and determine the volume, the term $\dot{V}/V$ is a function of only internal concentrations (see Equation 7 above). The time evolution of C_A and C_W within cortical condensates is therefore intensive as shown in the previous section.

Finally, in the main text we state that 'intensive condensate dynamics are not consistent with conventional kinetics of nucleation and growth of liquid-like condensates'. As an example of this, note that if WSP-1 were accumulating via diffusion limited nucleation and droplet growth from its supersaturated vapor, the WSP-1 self recruitment term would have the size-dependent form $\dot{W} \sim aW^{1/3} - b$ (equation 2.53 in reference[13]) with the constants a and b depending on thermodynamic parameters such as external diffusion constant, supersaturation and a capillary length. In this case, the cross terms $k_l \frac{AW}{V}$ and $k_b \frac{AW}{V}$ and depolymerization term would remain of mass action form and yet the time-evolution of C_A, C_W and ϕ would not be intensive.

Supplementary note 10: Requirements for achieving volume-independent condensate dynamics

We may generalize the discussion in section 6 to clarify the requirements for achieving intensive condensate dynamics. We consider an arbitrary scheme of chemical reactions involving n reactants generating a product in a container of varying volume V. The system is *well-mixed* if the system is effectively homogenized by diffusion on the shortest reaction time scale. This requires that the timescale for diffusion across the container is faster than any reaction. This requirement is satisfied when the container size is smaller than the typical length scale of inhomogeneities that can arise during the reaction timescale via reaction-diffusion coupling.

In this well-mixed regime, reaction rates are homogenous across all parts of the container. The rate of change of the number of molecules R_i with $i = 1, \ldots, n$ is in general a function of the

numbers of molecules of all species and the container volume. This can be expressed as $\dot{R}_i = f_i(R_1, R_2, \ldots, R_n, V)$. Since all reactions occur homogeneously in space, the reaction rate per volume $g_i = f_i/V$ is the same everywhere. The system therefore has a scaling property

$$f_i(R_1\xi, R_2\xi, \ldots, R_n\xi, V\xi) = \xi f_i(R_1, R_2, \ldots, R_n, V) \quad . \tag{18}$$

Choosing $\xi = 1/V$ reveals that reaction rates per volume $g_i(r_1, r_2...r_n)$ are functions of the concentrations $r_i = R_i/V$ only. We thus find that the reaction fluxes $\dot{R}_i$ are extensive and obey

$$\dot{R}_i = V g_i(r_1, r_2...r_n) \quad . \tag{19}$$

Since the volume $V(R_1, \ldots, R_n)$ depends on composition and therefore varies, we find

$$\dot{r}_i = \frac{\dot{R}_i}{V} - \frac{R_i\dot{V}}{V^2} = g_i(r_1, r_2, \ldots, r_n) - r_i\frac{\dot{V}}{V} \quad . \tag{20}$$

Thus intensive condensate dynamics will arise whenever $\dot{V}/V$ is itself intensive and independent of volume. This is equivalent to

$$\dot{V} = V h(r_1, r_2, \ldots, r_n) \quad . \tag{21}$$

Before determining the volume growth rate h we first note that V is extensive according to

$$V(R_1\xi, R_2\xi, \ldots, R_n\xi) = \xi V(R_1, R_2, \ldots, R_n) \quad . \tag{22}$$

Taking a derivative with respect to ξ and setting $\xi = 1$ we have

$$V(R_1, R_2, \ldots, R_n) = \sum_{i=1}^{n} R_i \frac{\partial V}{\partial R_i} \quad . \tag{23}$$

From equation 26 it follows that the effective molecular volumes

$$v_i(r_1, \ldots, r_n) = \frac{\partial V}{\partial R_i} \tag{24}$$

are intensive. Next, to determine the volume growth rate h that leads to intensive chemical dynamics we have $h = \dot{V}/V$ which yields

$$h = \frac{1}{V}\sum_{i=1}^{n} \frac{\partial V}{\partial R_i}\frac{dR_i}{dt} + \frac{1}{V}\sum_{i=1}^{n} R_i \frac{d}{dt}(\frac{\partial V}{\partial R_i}) \quad , \tag{25}$$

which simplifies to

$$h = \sum_{i=1}^{n} g_i v_i + r_i \dot{v}_i \quad . \tag{26}$$

equation 27 ensures that the sum $\sum_{i=1}^{n} r_i \dot{v}_i = 0$ giving,

$$h = \sum_{i=1}^{n} g_i v_i \quad , \tag{27}$$

Finally, using equation 24, we also obtain a rate of concentration changes.

$$\dot{r_i} = g_i - r_i h \quad . \tag{28}$$

When h is zero, that is for fixed volume systems, we recover conventional mass action kinetics of concentrations $\dot{r_i} = g_i$.

Note that the volume growth rate h and the concentration dynamics $\dot{r_i}$ are intensive if the system is well mixed with extensive total volume, and therefore both the volume fluxes and reaction fluxes are extensive.

Thus we learn, that while fixed volume containers need only be well-mixed in order to generate intensive chemical dynamics, a variable-volume container additionally requires that the rate of volume change be determined by an intensive per-volume generation rate (h). For cortical condensates this is true, because internal molecular amounts change via homogenous mass action kinetics and simultaneously determine the volume.

Supplementary References

1. Reich, J. D. *et al.* Regulated Activation of the PAR Polarity Network Ensures a Timely and Specific Response to Spatial Cues. *Current Biology* **29**, 1911–1923.e5 (2019).

2. Shelton, C. A. & Bowerman, B. Time-dependent responses to glp-1-mediated inductions in early C. elegans embryos. *Development* **122**, 2043–2050 (1996).

3. Tokunaga, M., Imamoto, N. & Sakata-sogawa, K. Highly inclined thin illumination enables clear single-molecule imaging in cells. *Nature Methods* **5**, 159–161 (2008). URL `https://doi.org/10.1038/nmeth1171`.

4. Berg, S. *et al.* ilastik: interactive machine learning for (bio)image analysis. *Nature Methods* **16**, 1226–1232 (2019).

5. Geisler, J., Victoria, T. Y., Grill, S. W. & Narayanan, A. *Springer Methods in Molecular Biology: Phase separated Biomolecular condensates - 'Mass Balance Imaging: A Phase Portrait Analysis for Characterizing Growth Kinetics of Biomolecular Condensates'* (2022, under consideration).

6. Tinevez, J.-Y. *et al.* TrackMate: An open and extensible platform for single-particle tracking. *Methods* **115**, 80–90 (2017).

7. Victoria, T. Y. & Narayanan, A. Dataset for: A condensate dynamic instability orchestrates actomyosin cortex activation (2022). URL `https://doi.org/10.17617/3.PIRFA2`.

8. Dimchev, V. *et al.* Induced arp2/3 complex depletion increases fmnl2/3 formin expression and filopodia formation. *Frontiers in Cell and Developmental Biology* **9** (2021). URL https://www.frontiersin.org/article/10.3389/fcell.2021.634708.

9. Mohamed, A. M. & Chin-Sang, I. D. The C. elegans nck-1 gene encodes two isoforms and is required for neuronal guidance. *Developmental Biology* **354**, 55–66 (2011).

10. Mitchison, T. & Kirschner, M. Dynamic instability of microtubule growth. *Nature* **312**, 237–242 (1984).

11. Dogterom, M. & Leibler, S. Physical aspects of the growth and regulation of microtubule structures. *Phys. Rev. Lett.* **70**, 1347–1350 (1993). URL https://link.aps.org/doi/10.1103/PhysRevLett.70.1347.

12. Fermi, E. *Thermodynamics* (1956).

13. Weber, C. A., Zwicker, D., Jülicher, F. & Lee, C. F. Physics of active emulsions. *Reports on Progress in Physics* **82**, 064601 (2019).
