# Supplementary material for: A condensate dynamic instability orchestrates actomyosin cortex activation
Source: Nature. 2022 Aug 17;609(7927):597–604. doi: 10.1038/s41586-022-05084-3 (PMC9477739; doi:10.1038/s41586-022-05084-3)
Supplement: Supplementary file 2 — Reporting Summary [file 41586_2022_5084_MOESM2_ESM.pdf]

Corresponding author(s): Stephan Grill

Last updated by author(s): 6/11/2022

Reporting Summary

Nature Portfolio wishes to improve the reproducibility of the work that we publish. This form provides structure for consistency and transparency in reporting. For further information on Nature Portfolio policies, see our Editorial Policies and the Editorial Policy Checklist.

Statistics

For all statistical analyses, confirm that the following items are present in the figure legend, table legend, main text, or Methods section.

n/a	Confirmed	
☐	☒	The exact sample size (*n*) for each experimental group/condition, given as a discrete number and unit of measurement
☐	☒	A statement on whether measurements were taken from distinct samples or whether the same sample was measured repeatedly
☒	☐	The statistical test(s) used AND whether they are one- or two-sided<br>*Only common tests should be described solely by name; describe more complex techniques in the Methods section.*
☐	☒	A description of all covariates tested
☐	☒	A description of any assumptions or corrections, such as tests of normality and adjustment for multiple comparisons
☐	☒	A full description of the statistical parameters including central tendency (e.g. means) or other basic estimates (e.g. regression coefficient) AND variation (e.g. standard deviation) or associated estimates of uncertainty (e.g. confidence intervals)
☒	☐	For null hypothesis testing, the test statistic (e.g. *F*, *t*, *r*) with confidence intervals, effect sizes, degrees of freedom and *P* value noted<br>*Give P values as exact values whenever suitable.*
☒	☐	For Bayesian analysis, information on the choice of priors and Markov chain Monte Carlo settings
☒	☐	For hierarchical and complex designs, identification of the appropriate level for tests and full reporting of outcomes
☒	☐	Estimates of effect sizes (e.g. Cohen's *d*, Pearson's *r*), indicating how they were calculated

Our web collection on statistics for biologists contains articles on many of the points above.

Software and code

Policy information about availability of computer code

Data collection	NIS Elements (2021),
Data analysis	Ilastik 1.4.0b14, Fiji (ImageJ 1.53C), Originlab version 6.1, Custom scripts in Python 3.8.5 and Matlab

For manuscripts utilizing custom algorithms or software that are central to the research but not yet described in published literature, software must be made available to editors and reviewers. We strongly encourage code deposition in a community repository (e.g. GitHub). See the Nature Portfolio guidelines for submitting code & software for further information.

Data

Policy information about availability of data

All manuscripts must include a data availability statement. This statement should provide the following information, where applicable:

- Accession codes, unique identifiers, or web links for publicly available datasets
- A description of any restrictions on data availability
- For clinical datasets or third party data, please ensure that the statement adheres to our policy

Source data are provided with this paper. Condensate track data are publicly available at https://doi.org/10.17617/3.PIRFA2

Human research participants

Policy information about studies involving human research participants and Sex and Gender in Research.

Reporting on sex and gender	N/A
Population characteristics	N/A
Recruitment	N/A
Ethics oversight	N/A

Note that full information on the approval of the study protocol must also be provided in the manuscript.

Field-specific reporting

Please select the one below that is the best fit for your research. If you are not sure, read the appropriate sections before making your selection.

☒ Life sciences ☐ Behavioural & social sciences ☐ Ecological, evolutionary & environmental sciences

For a reference copy of the document with all sections, see nature.com/documents/nr-reporting-summary-flat.pdf

Life sciences study design

All studies must disclose on these points even when the disclosure is negative.

Sample size	No sample size pre-calculation was performed. Each measured oocyte, contained sufficient condensate events (always >5000) to conduct the phase portrait analysis described in our manuscript and reach all the main conclusions. For comparison between the control and two RNAi datasets, data was combined from multiple oocytes in each condition. These sample sizes were chosen to ensure >100 condensates in each phase portrait bin in all three datasets - the number 100 was chosen to be well above that suggested by an analysis of the variance in phase portrait vectors..
Data exclusions	No data exclusions
Replication	All data and analysis results were replicable for multiple imaged C. elegans oocytes. Data from n oocytes in any given condition were combined for further analysis with n as stated across the manuscript and supplement.
Randomization	Worms and oocytes were randomly selected from growth plates corresponding to every experimental growth condition considered in this manuscript.
Blinding	Blinding is not applicable here, as experimental perturbations are performed by the same researcher as the measurement.

Reporting for specific materials, systems and methods

We require information from authors about some types of materials, experimental systems and methods used in many studies. Here, indicate whether each material, system or method listed is relevant to your study. If you are not sure if a list item applies to your research, read the appropriate section before selecting a response.

Materials & experimental systems

n/a	Involved in the study	
☒	☐	Antibodies
☒	☐	Eukaryotic cell lines
☒	☐	Palaeontology and archaeology
☐	☒	Animals and other organisms
☒	☐	Clinical data
☒	☐	Dual use research of concern

Methods

n/a	Involved in the study	
☒	☐	ChIP-seq
☒	☐	Flow cytometry
☒	☐	MRI-based neuroimaging

Animals and other research organisms

Policy information about studies involving animals; ARRIVE guidelines recommended for reporting animal research, and Sex and Gender in Research

Laboratory animals	C. Elegans, strains used listed in attached supplementary table 1.
Wild animals	Did not involve wild animals
Reporting on sex	N/A
Field-collected samples	This study did not involve Field-collected samples
Ethics oversight	Ethical oversight was not required for C. elegans research

Note that full information on the approval of the study protocol must also be provided in the manuscript.
